# The Impact of Surgical Margin Distance on Local Recurrence and Survival in Patients with Soft Tissue Sarcoma

**DOI:** 10.3390/medicina61020289

**Published:** 2025-02-07

**Authors:** Alparslan Yurtbay, Şafak Aydın Şimşek, Tolgahan Cengiz, Yakup Sancar Bariş, Ferhat Say, Nevzat Dabak

**Affiliations:** 1 Department of Orthopaedics and Traumatology, Samsun University, 55000 Samsun, Turkey; 2 Department of Orthopaedics and Traumatology, Ondokuz Mayıs University, 55139 Samsun, Turkey; drsafakaydin@gmail.com (Ş.A.Ş.); ferhatsay@gmail.com (F.S.); ndabak@gmail.com (N.D.); 3 Clinic of Orthopedics and Traumatology, Inebolu State Hospital, 37502 Kastamonu, Turkey; tolgahancengiz@hotmail.com; 4 Department of Pathology, Ondokuz Mayıs University, 55139 Samsun, Turkey; sancarbaris@omu.edu.tr

**Keywords:** orthopedic oncology, soft tissue sarcoma, surgical margin, neoplasm recurrence, disease-free survival

## Abstract

*Background and Objectives*: The primary objective of surgeons treating bone and soft tissue sarcomas (STS) is to achieve optimal local tumor control, ensuring a tumor-free margin and preventing local recurrence. However, the impact of surgical resection margin status on extremity STSs remains an area that requires further exploration. Therefore, this study aims to investigate the effects of surgical resection margin status on both local recurrence and overall survival rates. *Materials and Methods*: One hundred and eighty-five patients who underwent surgical resection with a diagnosis of soft tissue sarcoma were studied. The study recorded patient demographics, tumor characteristics, surgical margin distance (in millimeters), and disease-related outcomes. *Results*: The minimum follow-up period was 24 months (24–168). The mean time to local recurrence after resection was 103.2 months (95% CI 91.73 to 114.64). The mean local recurrence-free survival was found to be 7.23 months in patients with positive surgical margins, 87.42 months in patients with ≤1 mm, and 139.80 months in patients with >1 mm (*p* < 0.001). Patients with surgical margins ≤1 mm were more likely to have local recurrence than patients with >1 mm (0.41 [0.21–0.81], *p* = 0.010). The mean overall survival was 106.72 months (95% CI 95.98 to 117.46). Positive surgical margins were associated with decreased overall survival (3.58 [1.46–8.80], *p* = 0.005). There was a statistically significant difference between the histologic grade in terms of local recurrence (4.50 [95% CI 2.57 to 7.88]; *p* < 0.001) and overall survival (3.12 [95% CI 1.52 to 6.41]; *p* = 0.002). *Conclusions*: Achieving a negative surgical margin distance of more than 1 mm appears to be correlated with a reduced risk of local recurrence. Positive surgical margins are a risk factor that detrimentally impacts overall patient survival. However, determining the appropriate margin distance for all patients poses a significant challenge.

## 1. Introduction

Soft tissue sarcomas (STS) are a diverse group of heterogeneous malignancies that arise from mesenchymal cells with unique pathologic and clinical features. Sarcomas are rare, with about 16,250 cases annually in the U.S., comprising roughly 0.92% of all 1.76 million cancer diagnoses [1]. STS account for approximately 50% to 70% of all malignant tumors affecting the extremities [2]. There are over 100 histological subtypes of soft tissue tumors, mostly STS, according to the fifth edition of the WHO Classification of Tumors of Soft Tissue and Bone [3]. Managing these rare, heterogeneous tumors requires a multidisciplinary team of medical specialists.

Surgical resection is an essential part of a curative treatment plan for sarcoma management. The current protocols recommend a wide resection with negative microscopic margins. Surgery should prioritize limb-sparing techniques to preserve function [4,5]. Studies on the impact of margin status on the risk of recurrence in soft tissue sarcomas have yielded consistent findings [5,6,7,8]. The surgical management of soft tissue sarcoma benefits greatly from a collaborative, multidisciplinary approach. By fostering partnerships between orthopedic oncologists and plastic surgery teams in specialized referral centers, we can achieve optimal outcomes. This approach allows for the execution of wide oncologic resections while considering both effectiveness and the minimization of postoperative complications [9]. While various margin assessment methods have been suggested in orthopedic pathology, there is no universally accepted standard for determining an appropriate margin that effectively minimizes the risk of sarcoma-related complications such as local recurrence and patient survival [10].

Today, the concepts of clear surgical margins and positive surgical margins are still unclear. The Musculoskeletal Tumor Society classification was described by Enneking et al. and categorized surgical margins as intralesional, marginal, wide, and radical [11]. The American Joint Committee on Cancer employs a residual tumor classification system to determine the status of tumors after surgical resection [12]. The classification system distinguishes complete resections with microscopically negative margins as R0 resections, while incomplete resections with either microscopically or macroscopically residual tumors are classified as R1 and R2 resections, respectively. Further analyses have been conducted to investigate surgical margins using metric margin width [6,7,13,14,15].

Achieving negative margins is essential in surgical procedures, but determining the correct margin width is still challenging. There is no standard definition of an acceptable margin width for reducing adverse events related to sarcoma, leading to ongoing debate about the effectiveness of various margin metrics. This study will examine the impact of surgical margin distance on local recurrence and overall survival using a precise millimetric approach. The aim is to help develop clearer clinical guidelines for surgical margin distances in treating STS.

## 2. Materials and Methods

### 2.1. Study Design and Setting

This study was a retrospective, comparative analysis using data from a longitudinally maintained database at a single tertiary care university center. It is a Level of Evidence Level III therapeutic study.

### 2.2. Patients

**Figure 1:**
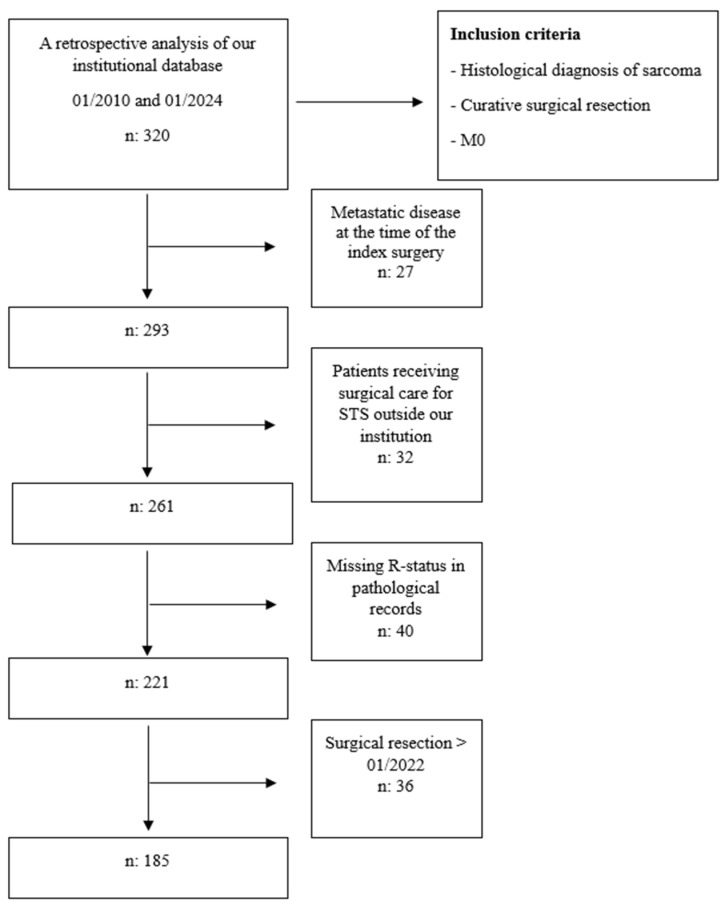
Flow diagram of inclusion and exclusion criteria.

We reviewed the medical records of 320 patients who received surgical treatment for STS from a specialized multidisciplinary team at a tertiary university hospital. This selection was made from a database of patients with bone and soft tissue tumors from January 2010 to January 2024. The patients included in the study were required to have a minimum of 2 years of follow-up. The excluded patients were those who had already developed metastases at the time of diagnosis, those who received unplanned surgical intervention for STS outside our clinic, those whose pathological records did not provide microscopic numerical data or R-status information, and those who underwent surgical resection after January 2022 (Figure 1). The study analyzed a cohort of 185 patients with localized STS in their extremities. The standard surgical approach involves en bloc excision with R0 margins, which means removing the tumor in a single specimen with a margin of normal tissue around it [16]. Surgical resection was performed on all sarcoma patients to achieve a negative margin, guided by multidisciplinary discussions based on histopathology and radiology reports. Tumor grades and surgical margins from pathologic reports were analyzed, and wide margins were achieved in all cases.

### 2.3. Pathology Protocol

The Federation of Turkish Pathology Societies Bone and Soft Tissue Pathology Guide, aligned with global standards, is used for macroscopic evaluation and sampling. Soft tissue tumor resections require precise margin marking with ink, accurate identification of previous surgical procedures, and preservation of surrounding anatomy. Tissue is sectioned into 1 cm slices, with sampling guidelines recommending at least one sample per centimeter of tumor diameter (a minimum of 20 tissue samples are required for tumors larger than 20 cm), and at least three samples should be taken from the closest margins and any margins close to anatomical structures such as nerves, blood vessels or bone. Lastly, taking samples from tumor areas exhibiting varying color, pattern, or consistency is essential. Microscopic evaluations for margins are expressed as positive (intralesional), marginal, or wide, per Enneking’s criteria. In the case of marginal resections, a stage millimetric grid is employed to provide the distance to the tumor in millimeters. Distances less than 1 mm are indicated as being closer than 1 mm.

### 2.4. Treatments

The treatment protocols for all the patients who participated in the study were designed after a comprehensive analysis of their radiology and histopathology reports during the multidisciplinary tumor board. This committee has been actively functioning at our hospital since 2004. To achieve negative surgical margins for sarcomas, all patients underwent resection.

Radiotherapy (RT) was given following a multidisciplinary evaluation of risk factors for local recurrence, which included consideration of surgical margins, tumor size, and histological type. It was a standard addition to surgery for high-grade (G2-3) lesions. However, if a tumor was completely encased within a single compartment, radiotherapy was not performed (20 patients). Out of 185 patients, 155 received adjuvant RT, 60 underwent chemotherapy, 50 received a combination of radiation therapy and chemotherapy, and 20 did not undergo additional treatment. Only 7 out of 185 patients underwent amputation surgery, whereas most (178 out of 185 patients) underwent extensive resection and limb salvage surgery. At the conclusion of the study period, 30 out of 185 patients died owing to disease-related complications. In total, 24 out of 185 patients died of factors not related to their disease (Table 1).

A significant correlation was identified between the types of tumors and the corresponding treatments (*p* < 0.001) (Table 1). The rate of those receiving CTX treatment in the UPS tumor type was 38.8%, that in the liposarcoma type was 19.2%, that in the myxofibrosarcoma type was 20%, that in the synovial sarcoma type was 35%, that in the fibrosarcoma type was 26.7%, that in the rhabdomyosarcoma type was 66.6%, that in the leiomyosarcoma type was 12.5%, and in other rare sarcoma types, this rate was 35.3%. The rate of those receiving RT treatment in the UPS tumor type was 90.3%, that in the liposarcoma type was 73.1%, that in the myxofibrosarcoma type was 92%, that in the synovial sarcoma type was 90%, that in the fibrosarcoma type was 80%, that in the rhabdomyosarcoma type was 75%, that in the leiomyosarcoma type was 62.5% and in other rare sarcomas, this rate was 76.4% (Table 1).

### 2.5. Variables

The database contains important data regarding the time it takes for local recurrence to develop, the time it takes for cancer to spread to other parts of the body (metastasis), any subsequent surgical procedures such as amputation or excision of local recurrence, as well as detailed information on the specific chemotherapy and radiation treatment regimens that were administered. Follow-up time was determined by measuring the time from the initial date of diagnosis to the date of the most recent follow-up visit or the date of death, if applicable.

We conducted a comprehensive analysis of clinical, pathologic, and treatment variables (age, gender, tumor location, tumor size, depth (superficial or deep about the fascia), grade, surgical margin distances, radiotherapy, chemotherapy, and local recurrence) regarding the clinical endpoints of local recurrence, overall survival, and local recurrence-free survival (LRFS). The histologic grade was determined using the French Federation Nationale des Centers de Lutte Contre le Cancer (FNCLCC) histological grading system [17].

The depth of the tumor was assessed preoperatively using magnetic resonance imaging (MRI) to determine whether it was located superficially or deeply based on its relationship to the fascia. Surgical margins such as the following were classified as an ordinal variable: microscopically positive (0 mm), no more than 1 mm wide, and more than 1 mm wide. While making this classification, the width ranges were narrowed further based on Dickinson et al. [18]. In cases where there was uncertainty regarding the positivity of the surgical margin during the surgical procedure, frozen section samples were obtained and sent to pathology for analysis. If the frozen section margin was positive, targeted resection was extended until a negative margin was achieved.

### 2.6. Primary and Secondary Study Outcomes

The primary outcome of our study aimed to evaluate local recurrence rates after surgical intervention, focusing on histologically defined surgical margins. We measured the distance between the resection surface and the nearest viable tumor, recording this in millimeters on histological slides [19,20]. The histologic status of the surgical margin was defined as microscopically positive (0 mm), negative (≤1 mm), and negative (>1 mm). Because Enneking et al. [11] defined marginal and wide excision as inherently subjective and suggested that it may vary depending on who is assessing the margin [21], we evaluated the width of the surgical margin in millimeters for this study [22].

Our secondary outcomes of interest comprised overall survival, disease-free survival (DFS), and LRFS, as well as their correlation with the FNCLCC histological grading system among patients who underwent surgical resection. DFS was defined as the time after primary treatment when patients with STS survived without symptoms. LRFS was defined as the time from the surgical procedure until the occurrence of local recurrence, which was censored at the latest follow-up visit or death. Overall survival was the period measured from the initial diagnosis of the disease until the patient’s death. We obtained patients’ survey-related data from national death registries. If there were any concerns regarding a potential local recurrence, it was thoroughly evaluated using MRI and subsequently confirmed histologically through a biopsy.

### 2.7. Ethical Approval

Ethical approval for this study was obtained from the Ondokuz Mayıs University clinical research ethics committee (approval number: 2023/477, date: 23 January 2024).

### 2.8. Statistical Analysis

The SPSS for Windows 23.0 program from SPSS Inc. was utilized for statistical analysis of the study data. Compliance with normal distribution was examined using Kolmogorov–Smirnov and Shapiro–Wilk tests. The Fisher–Freeman–Halton and Pearson Chi-Square test were used to examine categorical variables, and multiple comparisons were made with the Bonferroni-corrected Z test. The Kruskal–Wallis test was used to compare variables that did not comply with normal distribution in groups of three or more, and multiple comparisons were made with the Dunn test. The Kaplan–Meier test (log rank test) compared survival and recurrence times according to categorical variables. Cox regression analysis examined the risk factors affecting survival and recurrence times. Analysis results were presented as a frequency (percentage) for categorical variables and mean ± standard deviation and median (minimum–maximum) for quantitative variables. Statistical significance was set at a *p*-value of less than 0.05.

## 3. Results

### 3.1. Study Population

Of the 185 patients, 104 were male (Table 1). The median values of the patients’ ages according to tumor types showed a statistically significant difference (*p* < 0.001) (Table 2). The effect of gender was not associated with local recurrence (HR 1.08 [95% CI 0.60 to 1.94]; *p* = 0.79) (Table 3) and survival (HR 1.47 [95% CI 0.74 to 2.92]; *p* = 0.270) (Table 4). The median follow-up duration was 48 months (24–168). The median age of the patients at the time of diagnosis was 54 years (2–95). Age was not associated with local recurrence (HR 0.99 [95% CI 0.97 to 1.00]; *p* = 0.133) (Table 3) but associated with survival (HR 1.03 [95% CI 1.01 to 1.05]; *p* < 0.001) (Table 4). The median tumor dimension was 8 cm (2–36 cm). The mean time to local recurrence after resection was 103.2 months (95% CI 91.73 to 114.64) (Table 5). The mean overall survival time was 106.72 months (95% CI 95.98 to 117.46) (Table 6).

### 3.2. Relationship Between Margin and Local Recurrence

In total, 74 of the 185 patients experienced a local recurrence (Table 1). Seventy-seven percent of all local recurrences were observed within the first year. Overall, the 1-year local recurrence-free rates were 69.1% (95% CI, 48% to 89%). The cumulative probability of local recurrence within one year was 30.9%. Univariate analysis revealed that patients with surgical margins greater than 1 mm had a lower risk of recurrence compared to those with surgical margins equal to or less than 1 mm (HR: 0.34 [%95 CI 0.18 to 0.63], *p* = 0.001). Multivariate analysis confirmed that patients with surgical margins greater than 1 mm had a lower risk of recurrence compared to those with surgical margins equal to or less than 1 mm (0.49 [0.21–0.81], *p* = 0.010). The risk of recurrence in patients with positive surgical margins was 10.5 times higher than in those with surgical margins equal to or less than 1 mm (10.55 [5.26–21.15], *p* < 0.001) (Table 3).

**Figure 2:**
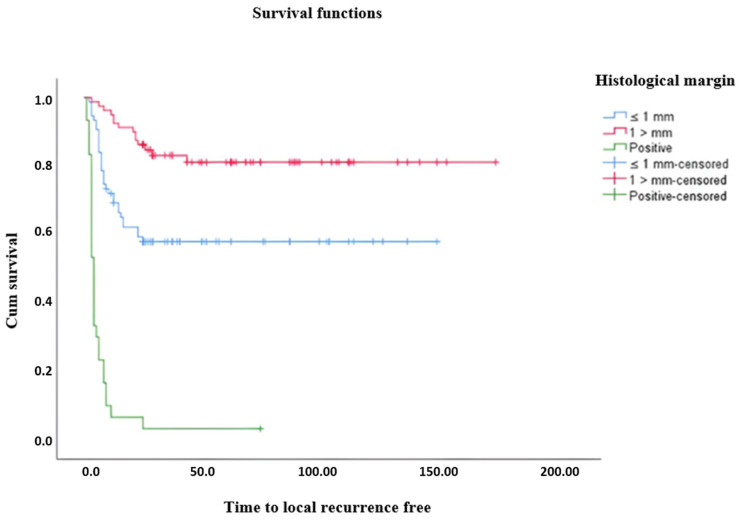
Kaplan–Meier curves showing local recurrence-free times and disease-free survival for patients grouped by histological margin who received surgical treatment for STS after wide resection.

A statistically significant difference was found between LRFS and surgical margin (*p* < 0.001) (Table 5). The mean LRFS was 87.42 months in patients with surgical margins equal to or less than 1 mm, 139.8 months in patients with margins greater than 1 mm, and 7.23 months in patients with positive surgical margins (Figure 2). The general LRFS average is 103.19 months.

### 3.3. Relationship Between Factors Other than Margin and Local Recurrence

Multiple risk factors affecting recurrence were examined by multivariate analysis. The risk of recurrence in patients with an undifferentiated pleomorphic sarcoma (UPS) tumor type was three times higher than in those with liposarcoma (2.98 [1.13–7.84], *p* = 0.027). The risk of recurrence in patients with the myxofibrosarcoma (MFS) tumor type was 3.7 times higher than in those with liposarcoma (3.7 [1.14–11.99], *p* = 0.029). The risk of recurrence in patients with the rhabdomyosarcoma tumor type was 7.4 times higher than in those with liposarcoma (7.4 [1.86–29.46], *p* = 0.005). The risk of recurrence in patients with other rare sarcoma tumor types was 5.4 times higher than in those with liposarcoma (5.42 [1.81–16.25], *p* = 0.003). The risk of recurrence in patients with a superficial depth above the fascia was lower than in those with a significant depth under the fascia (0.53 [0.30–0.91], *p* = 0.022). One unit increase in tumor grade was found to increase the risk of recurrence by 4.5 times (4.5 [2.57–7.88], *p* < 0.001). Other variables were not found to be a risk factor in recurrence (*p* > 0.050) (Table 3).

### 3.4. Relationship Between Margin and Overall Survival

Multivariate analysis revealed that those with positive surgical margins had a higher risk of death than those with surgical margins less than or equal to 1 mm (3.58 [1.46–8.80], *p* = 0.005). No statistical difference was found in terms of overall survival between those with surgical margins greater than 1 mm and those with equal to or less than 1 mm (1.30 [0.54–3.13], *p* = 0.553) (Table 4).

**Figure 3:**
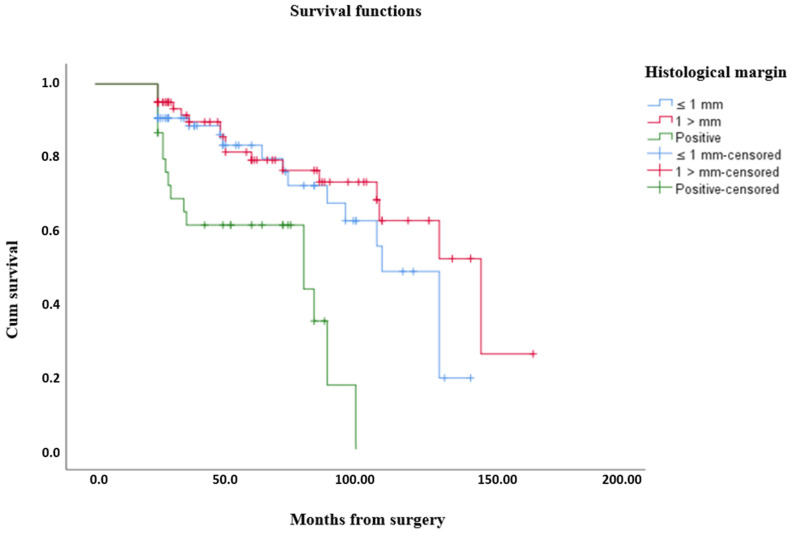
Kaplan–Meier curves show overall survival for patients grouped by histological margin who received surgical treatment for STS after wide resection.

A statistically significant difference was found between overall survival times according to surgical margins (*p* < 0.001) (Table 6). The average overall survival time was 103.95 months for those with a surgical margin equal to or less than 1 mm, 120.77 months for those with a margin greater than 1 mm, and 65.15 months for those with a positive surgical margin (Figure 3). The average overall survival time is 106.72 months.

### 3.5. Relationship Between Factors Other than Margin and Overall Survival

When the risk factors affecting overall survival were examined in a multivariate manner, the risk of death in patients with MFS tumor type increased 4.95 times compared to that in patients with liposarcoma (4.95 [1.21–20.20], *p* = 0.026). The risk of death in patients with rhabdomyosarcoma tumor type increased 8.95 times compared with that of those with liposarcoma (8.95 [2.04–39.29], *p* = 0.004). Those who received RT treatment had a lower risk of death than those who did not (0.32 [0.15–0.77], *p* = 0.009). As age increased, the risk of death increased 1.03 times (1.03 [1.02–1.05], *p* < 0.001). As the tumor grade increased, the risk of death increased 3.1 times (3.12 [1.52–6.41], *p* = 0.002). Other variables were not found to be a risk factor in survival time (*p* > 0.050) (Table 4).

### 3.6. Relationship Between Tumor Grade, Local Recurrence, and Overall Survival

According to our data, there was a statistically significant difference between histological tumor grades determined by the FNCLCC grading system and local recurrence (HR 4.50 [95% CI 2.57 to 7.88]; *p* < 0.001) (Table 3) and overall survival (HR 3.12 [95% CI 1.52 to 6.41]; *p* = 0.002) (Table 4).

## 4. Discussion

Soft tissue sarcomas are a heterogeneous group of mesenchymal tumors with over 100 subtypes, unique pathological and clinical features, and varying biological behaviors [3]. Treatment failures can manifest in various ways, including local recurrence, metastasis, and mortality. Multiple studies have indicated that positive margins following surgical procedures correlate with a heightened risk of local recurrence [23]. Due to the rarity, heterogeneity, and biological complexity of STS, more detailed clinical practice guidelines regarding surgical margin distance in treatment are lacking [20]. The relationship between microscopic surgical margin distances and local recurrence is still debated. How are local recurrence and overall survival affected as the distance from the tumor tissue increases? Is surgical margin width less important than achieving a negative margin at any distance? Answering these questions will contribute significantly to creating more detailed clinical practice guidelines regarding surgical margin distance in the surgical treatment of STS.

We examined microscopic surgical margins’ impact on local recurrence and overall survival in extremity STS patients. Our findings show that positive surgical margins are associated with decreased overall survival, while negative surgical margins greater than 1 mm predict reduced local recurrence compared to negative surgical margins less than 1 mm.

Positive surgical margins were associated with local recurrence compared to negative margins (less than 1 mm and greater than 1 mm). Beyond obtaining negative surgical margins, local recurrence was lower as the distance of the surgical margin from the tumor tissue increased. It was determined that the risk of local recurrence in patients with negative surgical margins greater than 1 mm was lower than that in patients with negative surgical margins less than 1 mm (*p* = 0.010) (Table 3). The ideal margin width for sarcoma resection that increases local control without undoing morbidity is controversial, as there is no commonly accepted standard-of-care margin distance [7,8,15,20,22,23,24,25,26] (Table 7).

Fujiwara et al. [15] measured the surgical resection margin as a metric distance. They reported that the wider the resection margin, the lower the risk of local recurrence. Gundle et al. [26] reported that the R + 1 mm classification reduced the local recurrence differences between R1 and R0 and that a negative but less than 1 mm margin may be adequate with multidisciplinary treatment. Kainhofer et al. [22] reported that resection margins with a minimum width of 1 mm seem appropriate to be designated negative resection margin status. Novais et al. [7] and Wittenberg et al. [25] defined surgical margins of 2 mm or less as inadequate surgical margins and associated them with local recurrence. Harati et al. [24] showed no difference in local recurrence at a median follow-up of 5 years when comparing different margin widths for negative margin resections. Ahmad et al. [27] emphasized achieving a negative margin to optimize local control and survival in patients undergoing RT and limb-sparing surgery for STS. However, they noted that the absolute quantitative width of the negative margin did not significantly affect the outcome, and therefore, attempts at extensive resection appeared unnecessary. However, the results obtained from this study should not be applied to patients who had surgery alone as a local treatment for their STS. They pointed out that wider resection margins may be necessary in this case. A surgical procedure with negative margins appears essential for reducing the likelihood of local recurrences and improving the survival of patients with recurrence after wide resection of STS [8]. Research indicates that obtaining negative microscopic surgical margins is vital for treating STS. Our analysis reveals that patients with positive surgical margins have a considerably greater risk of recurrence, particularly when margins measure 1 mm or less. In contrast, margins exceeding 1 mm demonstrate a lower risk. This highlights the significance of negative margins in minimizing local recurrence. Therefore, there is an urgent need for standardized guidelines concerning the appropriate width of resection margins.

Tumor-specific factors such as histological diagnosis and growth pattern should be considered when planning surgical treatment for STS. Some sarcoma subtypes, such as MFS and UPS, have an infiltrative growth pattern [28]. Even if negative surgical margins are achieved, MFS and UPS have reported a relatively high local recurrence rate compared with other STS subtypes [29,30]. When multiple risk factors affecting local recurrence were examined in our study, higher local recurrence rates were observed in UPS, MFS, and rhabdomyosarcoma tumor types compared with liposarcoma, which was selected as the control group (Table 3).

**Table 7:** Results of different surgical margin classifications on local recurrence for patients with STS.

Reference	Year	Number of Patients	Sarcoma Site	Margin Categories (mm)	Local Recurrence (%)	Follow-Up, Months	Comments
Fujiwara et al. [30]	2020	109	Low-grade	Positive Negative with a margin of 0.1–1.9 mm Negative with a margin ≥ 2.0 mm	38 8 0	Median: 67 (39–103)	While the negative margin resulted in 90% local control, excellent local control was achieved with margins of ≥2 mm.
Fujiwara et al. [15]	2020	305	High-grade nonmetastatic MFS or UPS	Positive 0.1 −0.9 1.0 −1.9 2.0 −4.9 5.0–9.9 >10	19 13 6 15 17 3	Minimum: 24.0	A margin distance of at least 10 mm is advocated for MFS and UPS to minimize the risk of LR.
Gundle et al. [26]	2018	2217	Nonmetastatic extremity and trunk STS	R + 1 mm classification Gross Positive (R2) <1 >1	38.0 10.0 6.0 (5-Year LR)	Mean 65 (0.3–309)	An R + 1 mm classification reduced LR differences between R1 and R0, suggesting that a negative but 1 mm margin may be adequate with multidisciplinary treatment.
Kainhofer et al. [22]	2016	113	All sites	Positive <1 ≥1	36.7 38.3 12.0	Mean: 68.5 (1.0–201.0)	Local control rates were superior after a minimal resection margin of 1 mm compared to R0 resections after the R-classification.
Harati et al. [24]	2017	643	Extremities	0.1 −0.9 1–5 ≥5	31.4 32.1 31.6	Median: 64.8	No difference in local recurrence when comparing different margin widths for negative margin resections.
Ahmad et al. [27]	2016	382	Low-to-high grade, extremity and truncal STS with R0 or R1 resection	Positive ≤1 >1–5 >5	83 LRFS 90 LRFS 93 LRFS 100 LRFS	Median: 82	The LRFS demonstrates superiority with any negative margin, yet there is no disparity in LRFS when comparing negative margin widths.
Novais et al. [7]	2010	248	Primary intermediate- high-grade STS of the extremity	≤2 2.1–20 >20	11.6 2.4 0.0	Median: 52.8 (4.8–156)	Margin > 2 mm recommended

LR = local recurrence; LRFS = local recurrence-free survival.

The risk of the local recurrence of deep, high-grade sarcomas may differ from that of low-grade tumors or subcutaneous sarcomas [31]. The detectability of subcutaneous sarcomas contributes to their potential for smaller size and lower malignancy. ’On the other hand, there is the potential for them to manifest as more significant and aggressive sarcomas. Subcutaneous sarcomas have a favorable prognosis, mainly determined by tumor-associated factors [32]. Their diagnostic and prognostic differences are significant when comparing deep-fascia sarcomas to subcutaneous sarcomas. This study supports existing knowledge, showing that the risk of recurrence is typically lower in superficial sarcomas above the fascia than in deeper ones (Table 3).

The standard high-grade tumor treatment involves wide excision and radiotherapy. The specific order of these treatments may vary among institutions. Still, there is a noticeable shift towards using preoperative RT, mainly when preserving a critical structure is a priority. Omitting RT is only considered after a comprehensive multidisciplinary discussion in specialized centers, considering several variables [4]. Studies have found that a high level of local control, ranging from 89% to 98%, can be achieved in treating STS. This success is attributed to the combination of wide resection and radiotherapy, which has reduced the need for amputation [33,34]. Additionally, radiotherapy for extremity STS has significantly improved the feasibility of limb salvage surgery [35]. The favorable impact of radiotherapy on reducing local recurrence observed in our study (Table 3), is consistent with findings in the existing literature.

The potential impact of chemotherapy on preventing local recurrence in STS is a subject currently sparking significant interest in the research community. For patients with STS who are at high risk for recurrence and/or metastasis, perioperative chemotherapy is a potential treatment option [36]. The role of chemotherapy in STS remains a matter of debate, and limited data are available on the specific effect of chemotherapy on margin status and local recurrence [20,37]. Gronchi et al. [38] have presented findings indicating that patients with margin-positive resection who underwent preoperative chemotherapy in conjunction with radiation exhibited a reduced likelihood of local recurrence compared to patients subjected to radiotherapy alone. Chemotherapy can help prevent the local recurrence of STS [39]. An experienced multidisciplinary sarcoma team makes the best decisions. Our study findings suggest no significant relationship between chemotherapy and the risk of local recurrence in the multivariate analysis conducted to understand the various risk factors influencing local recurrence. Additional research is needed to fully understand the effect of chemotherapy on local recurrence in localized STS cases.

The relationship between surgical margin status and overall survival in STS is crucial. Research shows that patients with positive margins are more likely to have local recurrence than those with negative margins, decreasing overall survival rates [6,7]. Patients with insufficient margins, such as positive margins or margins of 1 mm or less, may face an increased risk of local recurrence and potentially experience less favorable survival outcomes [40]. However, the influence of surgical margins on overall survival may vary, as some studies indicate that margin status may not directly predict overall survival or distant metastasis rates [41]. Achieving negative surgical margins is critical to reducing local recurrence. Still, factors such as clinical stage, tumor type, and histological grade also play an important role in determining overall survival in STS patients [40]. The data reveal a 5-year overall survival rate of 78.6%, consistent with previous studies. Our findings indicate that positive surgical margins are linked to decreased overall survival (*p* = 0.005). Additionally, we found no predicted difference in overall survival between negative surgical margins greater than 1 mm and negative surgical margins less than 1 mm (Table 4).

There is no consensus on the effect of the timing of RT on overall survival [33,34]. Therefore, we evaluated the entire RT group, considering preoperative and postoperative RT. Koshy et al. [42] reported that 47% of patients received radiotherapy, and no significant difference was observed in overall survival with radiotherapy in low-grade tumors. However, they noted that 3-year survival was higher in high-grade tumors (73%, 63%, *p* < 0.001). In multivariate analysis, radiotherapy increased survival in high-grade tumors (HR 0.67, 95% CI 0.57–0.79). Kungwengwe et al. [43] reported that preoperative radiotherapy was associated with wound problems, whereas postoperative radiotherapy provided a statistically significant survival advantage (n = 4192). In our study, 71% of patients who received radiotherapy were alive. When the risk factors affecting overall survival were examined in a multivariate manner, we showed that radiotherapy positively affected survival (*p* = 0.008).

Adjuvant chemotherapy is not a standard treatment for STS. However, it may be considered when there is a high risk of recurrence. The decision to pursue adjuvant chemotherapy should involve shared decision-making between the healthcare team and the patient, especially when there is uncertainty about its effectiveness. This is due to conflicting evidence from several decades of clinical trials despite meta-analyses being conducted [44]. Clinical trials have shown inconsistent results, with some large trials indicating no benefit from adjuvant and neoadjuvant chemotherapy. Conversely, smaller controlled trials and subgroup analyses of larger trials suggest that in cases where the risk of death is high, neoadjuvant or adjuvant chemotherapy may improve LRFS and overall survival [45,46,47]. In our study, 68.3% of patients receiving chemotherapy were alive, but no significant relationship was found between survival and chemotherapy (Table 4).

Extensive research on prognostic factors affecting local recurrence and overall survival in patients with extremity STS suggests that tumor grade is a particularly significant risk factor for patient survival [5,25,48]. Our data show a significant association between the histological tumor grades recorded using the FNCLCC grading system and the occurrence of local recurrence (Table 3) and overall survival (Table 4). Consequently, it is imperative to conduct stringent clinical follow-up evaluations for patients with pathologically high-grade tumors.

This study has several limitations. Margin determinations are based on pathology reports, which may lead to errors in tumor sampling. Differences in institutional practices for neoadjuvant and adjuvant treatments could affect outcomes, limiting the generalizability of the findings. Finally, the small number of patients who received neoadjuvant radiotherapy and chemotherapy prevents separate comparisons being made between these groups. Tissue types forming the surgical margin could not be analyzed. The type of tissue forming the surgical margin is as important as its width, and its impact on local recurrence and patient survival can be significant. Dense regular connective tissues, such as bone, periosteum, fascia, aponeuroses, and tendo ligamentous insertions, are all considered high-quality margins. In contrast, loose areolar fibrous tissue, muscle, and adipose tissue are of lower quality, thus requiring a larger margin distance for local control [20].

## 5. Conclusions

Evidence indicates that a negative surgical margin distance greater than 1 mm is correlated with a reduced incidence of local recurrence in patients compared to a negative margin distance of less than 1 mm. However, it does not appear to be correlated with overall patient survival. Tumor type, depth, and grade are important prognostic factors influencing local recurrence. Additionally, positive surgical margins, tumor type, radiotherapy, age, and tumor grade have been identified as important prognostic factors affecting overall patient survival. This research will significantly contribute to developing more comprehensive clinical practice guidelines regarding surgical margin distance in the surgical treatment of STS.

## Figures and Tables

**Table 1:** Examining the relationship between tumor type and categorical variables.

	Tumor Type								Test Statistics	*p*
	UPS	Liposarcoma	Myxofibrosarcoma	Synovial Sarcoma	Fibrosarcoma	Rhabdomyosarcoma	Leiomyosarcoma	Other Rare Sarcomas		
Sex										
Female	28 (45.2)	9 (34.6)	10 (40)	14 (70)	9 (60)	2 (16.7)	4 (50)	5 (29.4)	13.197	0.062 *
Male	34 (54.8)	17 (65.4)	15 (60)	6 (30)	6 (40)	10 (83.3)	4 (50)	12 (70.6)		
Tumor location										
Lower extremity	43 (69.4)	23 (88.5)	17 (68)	17 (85)	12 (80)	10 (83.3)	8 (100)	13 (76.5)	7.955	0.313 *
Upper extremity	19 (30.6)	3 (11.5)	8 (32)	3 (15)	3 (20)	2 (16.7)	0 (0)	4 (23.5)		
Histopathologic margin classification										
≤1 mm	28 (45.2)	9 (34.6)	9 (36)	11 (55)	4 (26.7)	5 (41.7)	5 (62.5)	4 (23.5)	12.643	0.533 *
>1 mm	26 (41.9)	12 (46.2)	12 (48)	5 (25)	7 (46.7)	7 (58.3)	2 (25)	9 (52.9)		
Positive	8 (12.9)	5 (19.2)	4 (16)	4 (20)	4 (26.7)	0 (0)	1 (12.5)	4 (23.5)		
Local recurrence										
No	38 (61.3)	18 (69.2)	17 (68)	12 (60)	7 (46.7)	8 (66.7)	4 (50)	7 (41.2)	5.799	0.567 *
Yes	24 (38.7)	8 (30.8)	8 (32)	8 (40)	8 (53.3)	4 (33.3)	4 (50)	10 (58.8)		
Metastasis										
No	54 (87.1)	26 (100)	21 (84)	15 (75)	14 (93.3)	10 (83.3)	7 (87.5)	14 (82.4)	8.737	0.212 *
Yes	8 (12.9)	0 (0)	4 (16)	5 (25)	1 (6.7)	2 (16.7)	1 (12.5)	3 (17.6)		
Depth										
Deep	35 (56.5)	12 (46.2)	16 (64)	11 (55)	6 (40)	3 (25)	5 (62.5)	8 (47.1)	7.184	0.404 *
Superficial	27 (43.5)	14 (53.8)	9 (36)	9 (45)	9 (60)	9 (75)	3 (37.5)	9 (52.9)		
Tumor grade										
1	2 (3.2) ^a^	6 (23) ^ab^	2 (8) ^ab^	1 (5) ^ab^	1 (6.7) ^ab^	3 (25) ^b^	1 (12.5) ^ab^	0 (0) ^ab^	21.02	0.040 *
2	28 (45.2)	12 (46.1)	10 (40)	7 (35)	5 (33.3)	4 (33.3)	3 (37.5)	9 (52.9)		
3	32 (51.6)	8 (30.7)	13 (52)	12 (60)	9 (60)	5 (41.7)	4 (50)	8 (47.1)		
Amputation										
No	57 (91.9)	26 (100)	24 (96)	19 (95)	15 (100)	12 (100)	8 (100)	17 (100)	3.851	0.781 *
Yes	5 (8.1)	0 (0)	1 (4)	1 (5)	0 (0)	0 (0)	0 (0)	0 (0)		
Prognosis										
DDU	12 (19.4)	3 (11.5)	2 (8)	3 (15)	1 (6.7)	1 (8.3)	2 (25)	0 (0)	15.26	0.297 *
DOD	10 (16.1)	1 (3.8)	5 (20)	4 (20)	1 (6.7)	4 (33.3)	2 (25)	3 (17.6)		
NED	40 (64.5)	22 (84.6)	18 (72)	13 (65)	13 (86.7)	7 (58.3)	4 (50)	14 (82.4)		
Adjuvant therapy ~										
CTX	24 (38.8) ^a^	5 (19.2) ^bc^	5 (20) ^b^	7 (35) ^c^	4 (26.7)	8 (66.6)	1 (12.5)	6 (35.3)	110.197	<0.001 **
RT	56 (90.3) ^ac^	19 (73.1) ^a^	23 (92)	18 (90) ^ab^	12 (80)	9 (75) ^b^	5 (62.5) ^bcd^	13 (76.4) ^ad^		

* Fisher–Freeman–Halton test; ** Pearson Ki-Kare test. ^a–d^: there is no difference between tumor types with the same letter. ~ multiple response; UPS: undifferentiated pleomorphic sarcoma.

**Table 2:** Comparison of quantitative variables according to tumor types.

	Age	Overall Survival	Local Recurrence Free Time	Tumor Size	Tumor Grade
Tumor type					
UPS (n = 62)	59.19 ± 19.98	51.81 ± 36.51	36.82 ± 38.35	9.92 ± 5.49	2.48 ± 0.5
	62.5 (14–86) ^cd^	33 (24–148)	24.5 (1–148)	8 (2–25)	3 (1–3)
Liposarcoma (n = 26)	46.16 ± 21.2	65.38 ± 33.69	47.23 ± 38.89	10.96 ± 7.48	2.08 ± 0.5
	45 (2–77) ^abc^	73 (24–120)	26.5 (3–109)	10.5 (3–36)	2 (1–3)
Myxofibrosarcoma (n = 25)	63.96 ± 14.96	52.08 ± 32.28	43.84 ± 39.44	10.8 ± 6.22	1.92 ± 0.65
	65 (35–85) ^bc^	39 (24–128)	34 (1–128)	10 (2–25)	3 (1–3)
Synovial sarcoma (n = 20)	47.5 ± 25.7	53.05 ± 30.03	34.05 ± 33.43	9.3 ± 5.82	2.5 ± 0.5
	45 (4–85) ^abc^	48 (24–132)	24 (2–110)	7 (3–26)	3 (1–3)
Fibrosarcoma (n = 15)	37.8 ± 16.48	71.07 ± 19.93	41.4 ± 32.01	7.27 ± 3.22	2.5 ± 0.51
	31 (23–73) ^a^	66 (48–110)	42 (3–108)	6 (4–14)	3 (1–3)
Rhabdomyosarcoma (n = 12)	19.91 ± 25.06	64.42 ± 54.63	52.58 ± 58.93	7.83 ± 2.25	2.08 ± 0.9
	7 (3–73) ^a^	36 (24–168)	26 (3–168)	8 (4–11)	2 (1–3)
Leiomyosarcoma (n = 8)	58.75 ± 25.59	60.5 ± 43.04	40.5 ± 38.94	9.75 ± 4.53	2.38 ± 0.52
	55 (24–95) ^abc^	43.5 (24–132)	24.5 (5–108)	8 (5–17)	3 (1–3)
Other rare sarcomas (n = 17)	41.24 ± 21.34	59.94 ± 36.78	31.53 ± 41.32	7.59 ± 5.41	2.47 ± 0.51
	46 (4–68) ^ad^	48 (24–144)	16 (2–144)	6 (3–22)	2 (2–3)
Test Statistics	39.186	9.863	4.669	9.019	5.300
*p* *	<0.001	0.196	0.700	0.251	0.623

* Kruskal–Wallis test; mean ± standard deviation; median (minimum–maximum); ^a–d^. There is no difference between tumor types with the same letter. UPS: undifferentiated pleomorphic sarcoma.

**Table 3:** Univariate and multivariate cox model analyses of prognostic factors for local recurrence.

	Local Recurrence		Univariate		Multiple	
	Negative	Positive	HR (%95 CI)	*p*	HR (%95 CI)	*p*
Tumor type						
Liposarcoma	18 (69.2)	8 (30.8)				
UPS	38 (61.3)	24 (38.7)	1.351 (0.607–3.007)	0.461	2.983 (1.134–7.842)	0.027
Myxofibrosarcoma	17 (68)	8 (32)	1.141 (0.428–3.042)	0.792	3.697 (1.14–11.987)	0.029
Synovial sarcoma	12 (60)	8 (40)	1.524 (0.572–4.062)	0.400	2.79 (0.879–8.855)	0.082
Fibrosarcoma	7 (46.7)	8 (53.3)	1.74 (0.653–4.637)	0.268	2.029 (0.67–6.151)	0.211
Rhabdomyosarcoma	8 (66.7)	4 (33.3)	1.08 (0.325–3.587)	0.900	7.403 (1.86–29.464)	0.005
Leiomyosarcoma	4 (50)	4 (50)	1.723 (0.519–5.722)	0.375	2.319 (0.57–9.444)	0.240
Other rare sarcomas	7 (41.2)	10 (58.8)	2.357 (0.929–5.976)	0.071	5.423 (1.809–16.25)	0.003
Sex						
Female	54 (66.7)	27 (33.3)				
Male	57 (54.8)	47 (45.2)	1.492 (0.929–2.396)	0.098	1.08 (0.601–1.942)	0.796
Tumor location						
Lower extremity	89 (62.2)	54 (37.8)				
Upper extremity	22 (52.4)	20 (47.6)	1.285 (0.769–2.147)	0.338	0.647 (0.358–1.169)	0.149
Histopathologic margin classification						
≤1 mm	44 (58.7)	31 (41.3)				
>1 mm	66 (82.5)	14 (17.5)	0.336 (0.178–0.632)	0.001	0.408 (0.206–0.808)	0.010
Positive	1 (3.3)	29 (96.7)	7.026 (4.099–12.042)	<0.001	10.552 (5.264–21.152)	<0.001
Depth						
Deep	46 (47.9)	50 (52.1)				
Superficial	65 (73)	24 (27)	0.405 (0.248–0.659)	<0.001	0.527 (0.304–0.913)	0.022
CTX						
No	62 (49.6)	63 (50.4)				
CTX	49 (81.7)	11 (18.3)	3.165 (1.829–6.135)	<0.001	1.165 (0.534–2.75)	0.687
RT						
No	22 (73.7)	8 (26.7)				
RT	89 (57.4)	66 (42.6)	2.361 (1.378–4.125)	0.005	1.414 (0.616–2.916)	0.584
Age	52.19 ± 22.8	49.16 ± 24.29	0.997 (0.987–1.007)	0.554	0.989 (0.975–1.003)	0.133
Tumor size	8.74 ± 4.73	10.76 ± 6.68	1.05 (1.015–1.088)	0.006	1.028 (0.982–1.076)	0.231
Tumor grade	2.3 ± 0.57	2.77 ± 0.42	3.926 (2.312–6.667)	<0.001	4.501 (2.57–7.883)	<0.001

**Table 4:** Univariate and multivariate cox model analyses of prognostic factors for overall survival.

	Survival		Univariate		Multiple	
	Alive	Dead	HR (%95 CI)	*p*	HR (%95 CI)	*p*
Tumor type						
Liposarcoma	22 (84.6)	4 (15.4)				
UPS	40 (64.5)	22 (35.5)	2.737 (0.932–8.031)	0.067	1.913 (0.585–6.262)	0.283
Myxofibrosarcoma	18 (72)	7 (28)	2.474 (0.722–8.484)	0.150	4.952 (1.214–20.201)	0.026
Synovial sarcoma	13 (65)	7 (35)	2.773 (0.806–9.544)	0.106	3.904 (0.997–15.283)	0.050
Fibrosarcoma	13 (86.7)	2 (13.3)	0.821 (0.15–4.498)	0.820	1.281 (0.218–7.51)	0.784
Rhabdomyosarcoma	7 (58.3)	5 (41.7)	2.124 (0.552–8.177)	0.273	8.946 (2.037–39.289)	0.004
Leiomyosarcoma	4 (50)	4 (50)	3.191 (0.792–12.865)	0.103	0.963 (0.159–5.83)	0.967
Other rare sarcomas	14 (82.4)	3 (17.6)	1.238 (0.276–5.542)	0.780	1.263 (0.262–6.079)	0.771
Sex						
Female	62 (76.5)	19 (23.5)				
Male	69 (66.3)	35 (33.7)	1.746 (0.996–3.062)	0.052	1.471 (0.74–2.925)	0.270
Tumor location						
Lower extremity	101 (70.6)	42 (29.4)				
Upper extremity	30 (71.4)	12 (28.6)	1.145 (0.597–2.196)	0.684	1.055 (0.507–2.194)	0.886
Histopathologic margin classification						
≤1 mm	55 (73.3)	20 (26.7)				
>1 mm	62 (77.5)	18 (22.5)	0.678 (0.355–1.297)	0.240	1.304 (0.543–3.131)	0.553
Positive	14 (46.7)	16 (53.3)	2.632 (1.328–5.218)	0.006	3.579 (1.456–8.798)	0.005
Local recurrence						
No	87 (78.4)	24 (21.6)				
Yes	44 (59.5)	30 (40.5)	2.67 (1.538–4.636)	<0.001	1.892 (0.866–4.135)	0.110
Depth						
Deep	68 (70.8)	28 (29.2)				
Superficial	63 (70.8)	26 (29.2)	0.96 (0.562–1.638)	0.880	1.077 (0.511–2.271)	0.846
CTX						
No	90 (72)	35 (28)				
CTX	41 (68.3)	19 (31.7.)	1.724 (0.914–3.745)	0.096	1.593 (0.675–3.882)	0.316
RT						
No	21 (70)	9 (30)				
RT	110 (71)	45 (29)	1.086 (0.645–1.956)	0.767	0.319 (0.154–0.765)	0.009
Age	45.81 ± 21.56	63.33 ± 23.13	1.028 (1.015–1.041)	<0.001	1.034 (1.015–1.053)	<0.001
Tumor size	8.66 ± 5.13	11.69 ± 6.34	1.047 (1.01–1.085)	0.012	1.019 (0.97–1.071)	0.452
Tumor grade	2.38 ± 0.57	2.74 ± 0.44	2.922 (1.65–5.173)	<0.001	3.121 (1.518–6.413)	0.002

**Table 5:** Comparison of local recurrence-free survival according to surgical margins.

	Mean (Months) (%95 CI)	Test Statistics	*p* *
Histopathologic margin classification			
≤1 mm	87.422 (72.201–102.643) ^c^	140.2	<0.001
>1 mm	139.799 (126.343–153.255) ^b^		
Positive	7.233 (2.665–11.802) ^a^		
Overall	103.187 (91.732–114.641)		

* Log rank test. ^a–c^: There is no difference between surgical margin groups with the same letter.

**Table 6:** Comparison of follow-up times (survival times) according to surgical margins.

	Mean (%95 CI)	Test Statistics	*p* *
Histopathologic margin classification			
≤1 mm	103.948 (91.217–116.679) ^b^	16.723	<0.001
>1 mm	120.771 (104.593–136.949) ^b^		
Positive	65.151 (53.011–77.291) ^a^		
Overall	106.722 (95.985–117.46)		

* Log rank test. ^a,b^: There is no difference between surgical margin groups with the same letter.

## Data Availability

The datasets used and/or analyzed during the current study are available from the corresponding author upon reasonable request.

## Abbreviations

The following abbreviations are used in this manuscript:

STS	soft tissue sarcomas
FNCLCC	the French Federation Nationale des Centers de Lutte Contre le Cancer
LRFS	local recurrence-free survival
DFS	disease-free survival
MRI	magnetic resonance imaging
UPS	undifferentiated pleomorphic sarcoma
MFS	myxofibrosarcoma
RT	radiotherapy
